# Therapeutic potential of neuroprotective plant extracts in Parkinson’s disease

**DOI:** 10.3389/fnins.2026.1813133

**Published:** 2026-04-01

**Authors:** Shifang Luo, Linao Zhang, Xue Wu, Jiying Wang, Qing Li, Wentao Chen, Rongyu Li, Lingli Zhou, Na Zhou, Rong Chen, Yuhuan Xie, Peixin Guo

**Affiliations:** 1 Yunnan Key Laboratory of Dai and Yi Medicines, Yunnan University of Chinese Medicine, Kunming, Yunnan, China; 2 College of Chinese Medicine, Yunnan University of Chinese Medicine, Kunming, Yunnan, China; 3 College of Basic Medical Sciences, Yunnan University of Chinese Medicine, Kunming, Yunnan, China

**Keywords:** antioxidant stress, anti-α-synuclein overexpression, mechanism of action, neuroprotective plant extracts, Parkinson’s disease

## Abstract

**Objective:**

This review aims to summarize the application and mechanisms of plant extracts with neuroprotective effects in Parkinson’s disease (PD), emphasizing their therapeutic potential in PD management.

**Background:**

Parkinson’s disease is a widespread neurodegenerative disorder, predominantly affecting middle-aged and elderly populations. Characterized by varied etiologies, clinical presentations, and complex pathogenesis, its hallmark symptoms include tremor and bradykinesia. Despite the availability of limited pharmacological treatments, current approaches primarily focus on symptom management rather than modifying disease progression. Recent research have increasingly investigates the use of neuroprotective plant extracts, which have shown demonstrating promising therapeutic effects, garnering significant attention in the field.

**Methods:**

A systematic review was conducted on preclinical and clinical studies published from 2000 to 2025, sourced from PubMed, ScienceDirect, Google Scholar, and China National Knowledge Infrastructure (CNKI), to evaluate the effects of neuroprotective plant extracts in PD treatment.

**Results:**

The review reveals that plant extracts with neuroprotective properties exert anti-PD effects through mechanisms including antioxidant and anti-inflammatory actions, inhibition of α-synuclein (α-syn) aggregation, enhancement of dopaminergic neuron survival, and restoration of synaptic function.

**Conclusion:**

This review outlines research directions for the development of neuroprotective plant extracts as novel PD therapies, suggesting their potential as complementary treatments alongside conventional pharmacological interventions.

## Graphical Abstract

**Figure:**
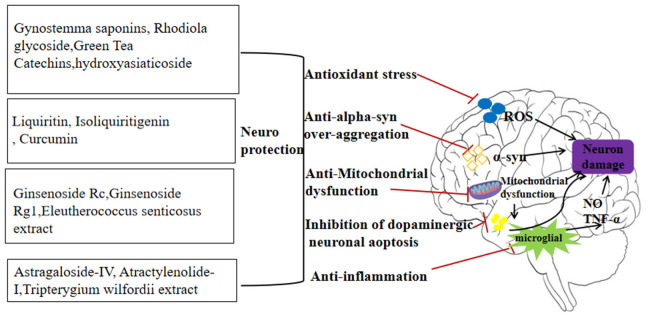
α-Syn, Anti-α-synuclein; ROS, reactive oxygen species; NO, Nitric oxide; TNF-α, Tumor necrosis factor-alpha.

## Highlights

1. Plant extracts with neuroprotective properties can markedly alleviate motor impairments in patients with Parkinson’s disease, reducing key symptoms such as tremors and bradykinesia.
2. Neuroprotective plant extracts exert their effects through multi-target, multi-pathway, providing protection by attenuating inflammation, inhibiting excessive α-synuclein (α-syn) aggregation, reducing oxidative stress, and preserving essential processes such as synaptic plasticity, neurotransmitter synthesis and release, and neuronal survival. These characteristics present substantial therapeutic potential for Parkinson’s disease management.

## 1 Introduction

Parkinson’s disease (PD) is a progressive neurodegenerative disorder marked by tremors and bradykinesia. As the second most prevalent neurodegenerative disease globally, it manifests with motor symptoms such as resting tremor, bradykinesia, rigidity, and postural instability (Jiang et al., 2024), alongside non-motor symptoms including anxiety, depression, sleep disturbances, cognitive impairment, and gastrointestinal dysfunction. Pathologically, PD is characterized by pigment loss in the substantia nigra and locus coeruleus, neuronal loss in the substantia nigra pars compacta, and dopamine depletion (Hayes, 2019; Yoo et al., 2024). The degeneration of dopaminergic neurons is considered central to its pathogenesis (Tai et al., 2024), with additional research implicating inflammation, mitochondrial dysfunction, and oxidative in disease progression (Matsui and Takahashi, 2024; Liu et al., 2024). Abnormal abnormal accumulation of α-synuclein (α-syn) and disturbances in mitochondrial function, autophagy, and apoptosis further contribute to PD’s pathological mechanisms. Immunotherapy strategies targeting α-syn clearance to reduce brain deposition, with potential improvements in cognitive function, are under investigation (Han et al., 2024; de Boni et al., 2024). While aging is the primary risk factor, environmental and genetic factors also play a role in modulating disease risk and progression (Shadrina and Slominsky, 2023).

Current clinical treatments for PD are based on its pathophysiology, with therapies including dopamine receptor agonists (e.g., levodopa, pramipexole), anticholinergic drugs (e.g., benztropine), and monoamine oxidase inhibitors (MAOIs) (e.g., rasagiline) (Mahlknecht and Poewe, 2024; Xie et al., 2024). Levodopa, which crosses the blood-brain barrier serves as an exogenous precursor to replenish dopamine in surviving neurons, effectively alleviating symptoms. Although it remains the most effective treatment, prolonged use can lead to disabling motor complications and does not modify disease progression, with potential adverse effects on the central nervous system (Zhang et al., 2024). Benztropine, an anticholinergic, enhances dopamine action by antagonizing central striatal M-cholinergic receptors. While it was the first drug used in PD treatment, chronic use can lead to side effects such as confusion, memory deficits, attention problems, and psychiatric issues (Yasaman et al., 2021). Rasagiline, an MAOI, inhibits monoamine oxidase activity to prevent dopamine breakdown, increasing dopamine levels in the brain. Though safe and effective in early-stage PD treatment, it can still cause side effects such as dizziness, nausea, vomiting, lightheadedness, and syncope (Yasaman et al., 2021).

The pursuit of innovative drug development and more effective approaches for preventing and treating PD has remained a central focus in pharmaceutical research. Exploring traditional medicine for potential therapeutic agents has emerged as a promising avenue for drug discovery. Medicinal plants with neuroprotective properties offer valuable resources for treating central nervous system disorders. Pharmacological studies indicate that extracts from these plants exert therapeutic effects against PD through mechanisms such as antioxidant and anti-inflammatory actions, inhibition of α-syn aggregation, preservation of dopaminergic neuron survival, and restoration of synaptic function (Xinhong et al., 2021). Neuroprotective plant extracts present distinct advantages in managing neurological disorders. Combining modern pharmacological treatments with neuroprotective plant extracts may provide a viable strategy for PD management. A literature search was conducted to evaluate the current status of neuroprotective plant extracts in PD treatment, a literature search of studies published between 2000 and 2025 in PubMed, ScienceDirect, Google Scholar, and CNKI was conducted. Keywords such as “Parkinson’s disease,” “neuroprotective plants,” and “neuroprotective drug compounds” were used. Inclusion criteria: ➀ Study type: randomized controlled trials, case-control studies, or basic research (including *in vitro* cell experiments and *in vivo* animal models); ➁ Study subjects: PD patients meeting internationally recognized diagnostic criteria (e.g., UK Brain Bank criteria), PD animal models (e.g., MPTP- or 6-OHDA-induced models), or PD-related cell models; ➂ Intervention: the experimental group received treatment with neuroprotective plant extracts or plant-derived monomeric compounds; Based on the exclusion criteria: ➀ Articles without the full text were excluded; ➁ Studies that were duplicates or from which data could not be extracted were screened out. This review aims to summarize the latest advancements in the clinical application and mechanistic studies of neuroprotective plant extracts in PD, highlighting their therapeutic potential and laying the groundwork for their clinical implementation and further drug development.

## 2 Clinical application of neuroprotective plants and their extracts in anti-Parkinson’s disease therapy

As PD progresses, patients experience a gradual decline in motor function, including slowing of movement, tremors in the hands, feet, or other body parts, and a loss of flexibility leading to stiffness. These symptoms tend to worsen with age, with tremors becoming more severe. Current clinical research supports the use of neuroprotective drugs in combination with conventional medications to enhance treatment outcomes for patients with PD.

In a study involving 128 patients with moderate-to-late-stage PD, participants were randomly assigned to four groups. The control group received standard levodopa (6.25 mg/kg), while the other three groups received levodopa combined with varying doses of *Astragalus* decoction (0.5, 1, or 2 g/kg). After 8 weeks of treatment period, the addition of *Astragalus* decoction significantly reduced scores on the Unified Parkinson’s Disease Rating Scale (UPDRS) Part III and the Parkinson’s Disease Quality of Life Questionnaire (PDQ-39) compared to levodopa monotherapy. Furthermore, it resulted in a more pronounced reduction in TNF-α and IL-1β levels, with efficacy positively correlated to the dose of *Astragalus* administered (Tong, 2023). Another study involving 30 Cases of Mid-Stage Tremor-Dominant with PD combined dopamine agonist tablets (0.0125 g/kg) with a modified Zhi Gan Cao Tang decoction (5.035 g/kg). This decoction, composed of raw *Rehmannia* root, *Ophiopogon* tuber, ginseng, roasted licorice, cinnamon twig, jujube, dried plum, silkworm pupae, raw gypsum, *Stephania* root, processed aconite root, charred ginger, *Schisandra* berry, and wine-processed cornelian cherry in a 50:12:6:12:7:30:3:3:6:6:6:6:2:2 ratio, showed therapeutic effects by intervening in α-syn oligomer production and disrupting its transport via microtubule pathways *in vivo* (Yifeng et al., 2024). In a study with 28 cases of early-stage primary with PD, combined treatment with *Acanthopanax* senticosus water extract (0.03 g/kg/day) and levodopa (0.01 mg/kg) over 3 months resulted in significant improvements in the Motor Function Rating Scale for Parkinson’s Disease (MDRSPD) score (Lei et al., 2018). A study involving 60 PD individuals with insomnia found that treatment with Ginseng Yangrong Decoction (1.57 g/kg) combined with repetitive transcranial magnetic stimulation (rTMS) for 4 weeks resulted in significant improvements in sleep quality, PD symptoms, and serum levels of serotonin (5-HT), norepinephrine (NE), and brain-derived neurotrophic factor (BDNF) (Lei et al., 2018). Additionally, a study of 39 patients with early-stage with PD showed that treatment with oral licorice syrup (0.2 mg/kg) combined with praloxerine (0.360 mg/day) for 24 weeks resulted in sustained improvement in UPDRS total scores, activities of daily living, and tremor symptoms. This regimen did not induce significant electrolyte disturbances or metabolic abnormalities (Lei et al., 2018; Table 1).

**TABLE 1:** Clinical applications of neuroprotective plants in PD.

Drug composition	Dosage administration	Combination therapy and dosage	Number of patient	Treatment course	Key findings	References
*Astragalus* decoction	0.5, 1, 2 g/kg	Madopar (6.25 mg/kg)	128 patients with moderate-to-late-stage Parkinson’s disease	8 weeks	UPDRS-III Score (based on tremor, rigidity, and bradykinesia) ↓, TNF-α↓, and IL-1β↓)	Tong, 2023
Modified fried licorice decoction (composed of raw *Rehmannia* root, *Ophiopogon* tuber, ginseng, fried licorice root, cinnamon twig, jujube, black plum, stiff silkworm, raw gypsum, *Stephania* root, processed aconite root, charred ginger, *Schisandra* berry, and wine-processed cornus fruit, with the following proportions: 50:12:6:12:7:30:3:3:6:6:6:6:2:2)	5.035 g/kg	Dopamine hydrochloride tablets (0.0125 g/kg)	30 cases of Mid-Stage Tremor-Dominant Parkinson’s disease	12 weeks	α-synuclein oligomers↓	Yifeng et al., 2024
*Eleutherococcus senticosus*	0.03 g/kg	Methadopa (0.01 mg/kg)	28 cases of early-stage primary Parkinson’s disease	12 weeks	MDRSPD score ↓ (based on resting tremor, limb rigidity, posture, facial expression, upper limb accompanying swing, writing, standing up, gait, speech, and standing stability)	Lei et al., 2018
Ginseng nourishing decoction (composed of ginseng, *Astragalus*, white *Atractylodes*, prepared *Rehmannia* root, poria, white peony root, cinnamon bark, fried polygala root, angelica root, *Schisandra* berries, dried tangerine peel, and licorice root, with a drug ratio of 10:10:10:10:10:10:10:10:9:9:6:6)	1.57 g/kg	Undergo rTMS treatment	60 patients with insomnia-type Parkinson’s disease	12 weeks	Tremor↓, 5-HT↑, NE↑, BDNF↑, total sleep time↑, sleep latency↓, arousal episodes↓, arousal time↓, sleep efficiency ↑	Haiyan et al., 2025
Licorice syrup	0.2 mg/kg	Prasugrel (0.360 mg/day)	39 patients with early-stage Parkinson’s disease	24 weeks	Total UPDRS↓ (scored based on speech, facial expression, resting tremor, sleep, posture, and general bradykinesia)	Petramfar et al., 2020

The clinical studies discussed above provide preliminary evidence for the therapeutic potential of neuroprotective plant extracts in PD treatment. These investigations indicate that neuroprotective plants and their formulations offer promising therapeutic potential through multi-target mechanisms, such as modulating α-syn aggregation, reducing neuroinflammation, and enhancing motor function. However, several significant limitations persist in current research: first, many studies combine neuroprotective plants with modern drugs, complicating the assessment of the plant extracts’ independent efficacy. Second, considerable variability exists in dosage selection, treatment duration, and efficacy assessment criteria across studies, highlighting the absence of standardized protocols. Therefore, future research should focus on more rigorous randomized controlled trials to systematically evaluate the independent therapeutic effects of neuroprotective plant extracts and further elucidate their mechanisms of action, thereby providing more reliable evidence to support their clinical application.

## 3 Neuroprotective plant extracts in preclinical anti-PD effects

### 3.1 Antioxidant stress

The central nervous system is particularly susceptible to oxidative stress due to its high metabolic activity and oxygen demands (Bhattacharya et al., 2024). Oxidative stress arises when there is an imbalance between the production or accumulation of reactive oxygen species (ROS) and the body’s intrinsic antioxidant defenses, leading to irreversible cellular and tissue damage (Suzuki and Bono, 2024). Excessive ROS can damage mitochondrial structures components, including membrane lipids, proteins, and mitochondrial DNA (mtDNA), and disrupt electron transport chain function, ultimately causing mitochondrial dysfunction. This mitochondrial damage results in ATP depletion and calcium influx, triggering apoptosis. Conversely, mitochondrial dysfunction exacerbates oxidative stress by generating uncontrolled ROS, initiating a vicious cycle that leads to cellular injury, organ failure, and disease progression (Gahtani et al., 2024).

Neuroprotective plant extracts possess potent antioxidant and free radical scavenging properties, with their mechanisms likely linked to the activation of the Nrf2 signaling pathway and modulation of ROS production within mitochondria. *Gynostemma saponins* (400 mg/kg, administered for 42 days) demonstrate significant protective effects in a rotenone-induced PD rat model. These effects include enhanced antioxidant activity of superoxide dismutase (SOD) and increased brain dopamine levels. Immunohistochemical analysis revealed a significant increase in the number of tyrosine hydroxylase (TH)-positive neurons in the substantia nigra pars compacta of treated rats. Further studies showed that *Gynostemma saponins* also upregulate DJ-1 protein expression, stabilizing Nrf2 protein and boosting cellular antioxidant defense (Jiaxuan et al., 2011). From a structure-activity perspective, the antioxidant and anti-apoptotic activities of *Gynostemma saponins* are directly correlated with the oxidative modifications on their aglycone side chains. Research indicates that the introduction of epoxy groups primarily inhibits apoptosis by stabilizing mitochondrial function, while functional groups such as carboxyl, carbonyl, or hydroxyl groups act as “secondary pharmacophores,” significantly enhancing free radical scavenging efficiency through electron delocalization effects (Jiaxuan et al., 2011).

The protective effect of rhodioloside, the primary component of *Rhodiola crenulata* (Hook. f. et Thoms.) H. Ohba, against MPP^+^-induced damage in MN9D cells has been confirmed by several studies. Experiments showed that rhodioloside treatment (10, 25, 50 μM, administered 24 h prior) increased cell survival rates while reducing intracellular ROS levels. Western blot analysis revealed a significant upregulation of DJ-1 protein expression and promoted Nrf2 nuclear translocation. Knockdown of DJ-1 or Nrf2 expression was knocked down using siRNA, the protective effect of rhodioloside on MPP^+^-induced MN9D cell viability decline was eliminated, indicating a strict dependence on the Nrf2 pathway. These studies suggest that rhodioloside enhances cellular antioxidant defense by regulating the Nrf2 pathway (Li et al., 2019). From a structure-activity relationship perspective, the antioxidant capacity of rhodioloside is intrinsically linked to its phenolic hydroxyl structure. Acting as an efficient hydrogen donor, the phenolic hydroxyl group effectively scavenges ROS, such as peroxyl radicals, converting itself into a less reactive radical intermediate. This characteristic forms the molecular basis for its ability to mitigate oxidative stress and protect cells from damage (Li et al., 2019).

Green Tea Catechins (GTCs) (2 mg/kg, administered for 14 days) demonstrated significant neuroprotective effects in a 6-hydroxydopamine (6-OHDA)-induced PD rat model. GTCs notably increased striatal superoxide dismutase (SOD) content and elevated glutathione peroxidase (GSH-Px) levels, enhancing the body’s antioxidant defense. Additionally, GTCs activate the Nrf2/ARE antioxidant signaling pathway by promoting Nrf2 nuclear translocation, upregulating heme oxygenase-1 (HO-1) expression, and downregulating Kelch-like epoxychloropropane-1-protein (Keap1) expression (Jianqin and Jianchen, 2017). In a study of transgenic A53T α-syn mice, GTCs (50 mg/kg/day, administered for 90 days) significantly improved motor coordination and anxiety-related behaviors. GTCs prolonged the latency period in the rotarod test, reduced plasma α-syn levels, and upregulated DJ-1 protein expression in brain tissue. Furthermore, they promoted Nrf2 nuclear translocation and inhibited microglial activation (Riegelman et al., 2024).

**FIGURE 1:**
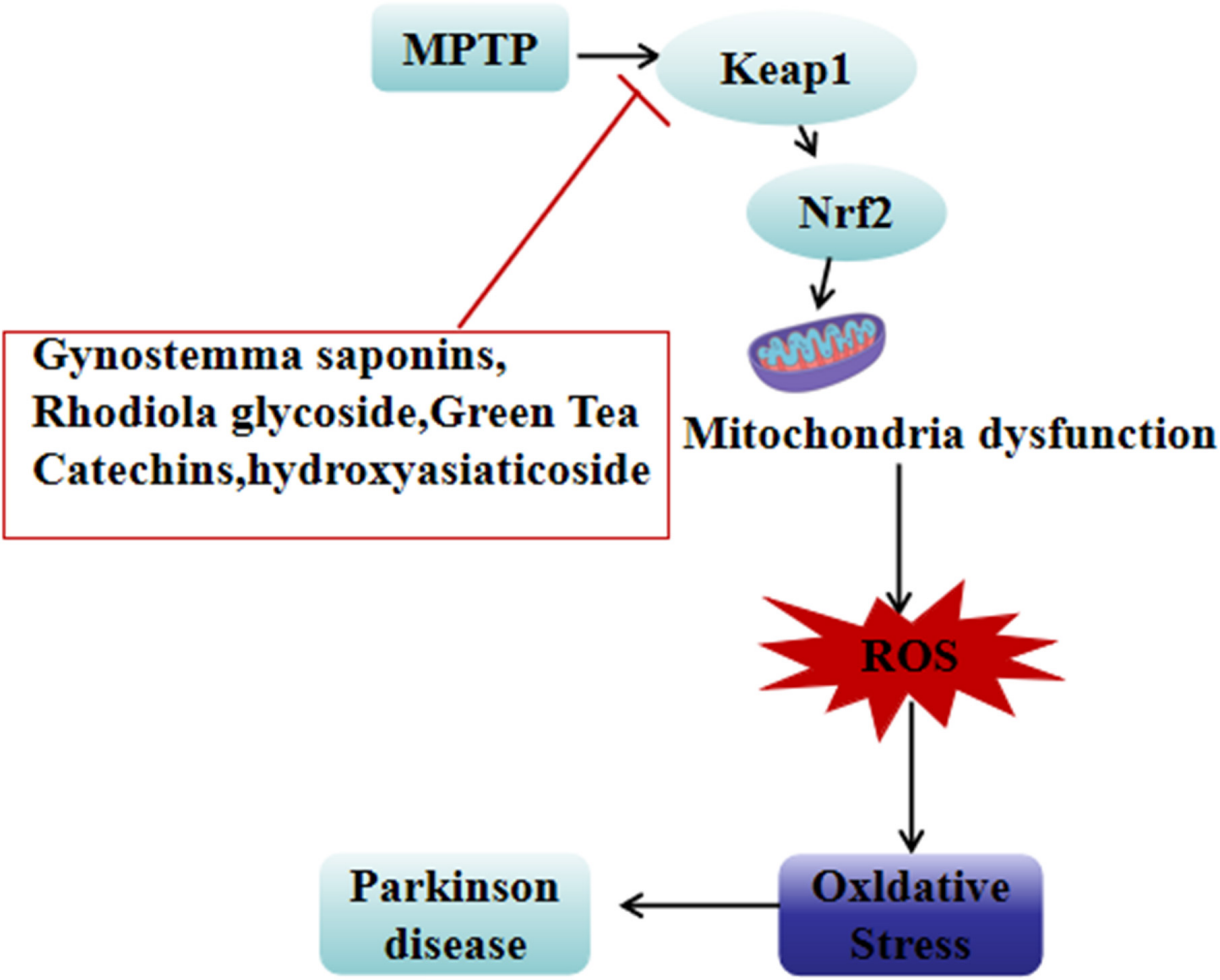
Neuroprotective plant extracts against oxidative stress. ⊥, inhibit; ↓, reducing; Nrf2, nuclear factor erythroid 2-related factor 2; MPTP, mitochondrial permeability transition pore; Keap1, Kelch-like ECH-associated protein 1; ROS, reactive oxygen species.

The primary active component of *Centella asiatica* (L.) Urb., hydroxyasiaticoside (2 mg/kg/day, administered for 14 days), exhibits significant multi-target neuroprotective effects in a rotenone-induced PD rat model. Behavioral tests showed significant improvements in rats treated with hydroxyasiaticoside, with enhanced suspension test scores. This compound also markedly increased striatal dopamine levels and boosted SOD activity. Mechanistic studies revealed that hydroxyasiaticoside significantly reduced abnormal α-syn aggregation by regulating protein L-isocysteine methyltransferase (PIMT) function. Additionally, it activated the AMPK/SIRT1 signaling pathway and upregulated Nrf2 nuclear translocation, thereby enhancing the body’s antioxidant defense capacity (Lanyue, 2020; Figure 1 and Table 2).

**TABLE 2:** Application of neuroprotective plant extracts in experimental studies.

Drug ingredients	Source	Model	Treatment	Key findings	References
*Gynostemma saponins*	*Gynostemma pentaphyllum*	Intraperitoneal injection of rotenone (1 mg/kg) induced a PD rat model, administered continuously for 42 days.	Daily oral administration of *Gynostemma pentaphyllum* (400 mg/kg) for 42 consecutive days	SOD↑, DA↑, number of TH-positive neurons↑, DJ-1↑, Nrf2↓	Jiaxuan et al., 2011
*Rhodiola glycoside*	*Rhodiola*	Establishment of a PD cell model by treating MN9D cells with MPP+ (200 μM) for 24 h	Cells were pretreated with rhodiololide (10, 25, 50 μM) for 24 h, followed by a 24-h treatment with MPP (200 μM).	ROS↓, DJ-1↑, Nrf2↓	Li et al., 2019
Green tea polyphenols	Green tea	A rat model of Parkinson’s disease was generated by unilateral striatal destruction using 6-OHDA (0.096 mg/kg).	Administration of 400 mg/kg of green tea polyphenols daily *via* gastric gavage for 6 consecutive weeks.	SOD↑, GSH-Px↑, Nrf2↓, HO-1↑, Keap1↓	Jianqin and Jianchen, 2017
Green Tea Catechins	Green tea	Transgenic A53T α-synuclein mice	Administration of 50 mg/kg/day of Green Tea Catechins daily for 90 days.	α-syn↓, DJ-1↑, Nrf2↓	
Hydroxyproline	*Centella asiatica*	Intraperitoneal injection of MPTP (27 mg/kg) to induce a PD mouse model, administered for 5 consecutive days.	Intraperitoneal injection of hydroxycentella glycoside (2 mg/kg), once daily, for 14 days.	DA↑, SOD↑, α-syn↓	Lanyue, 2020

### 3.2 Anti-α-synuclein overexpression

The pathological hallmark of PD is the excessive aggregation of α-syn. Consequently, reducing abnormal α-syn deposits is a critical focus in the development of anti-PD drugs (Negi et al., 2024). α-syn acts as both a primary marker and a central component in the pathogenesis of PD, representing a key target for genetic influences and functioning as a connector across various pathogenic mechanisms. Both the oligomerization and excessive aggregation of α-syn contribute to the onset and progression of the disease (He et al., 2024). In terms of neurotoxic mechanisms, α-syn oligomers promote pathological progression by inducing dopaminergic neuron death through multiple pathways, including disruption of cell membrane integrity, mitochondrial function, lysosomal stability, and cytoskeletal homeostasis. Additionally, excessive α-syn aggregation activates microglia and astrocytes, triggering the release of pro-inflammatory factors that exacerbate neuroinflammation. Furthermore, α-syn aggregation induces the production of excessive ROS, thereby triggering oxidative stress responses.

Experimental studies indicate that licorice components, specifically isoliquiritinide and glycyrrhizin, play a dual role in regulating the pathological aggregation of α-syn. *In vitro* experiments demonstrate that isoliquiritinide reduces oligomer formation, while glycyrrhizin inhibits fibrillation. In therapeutic contexts, isoliquiritinide (250 μM) has demonstrated fibril-disaggregating properties, reducing the aggregate size formed during α-syn aggregation after 72 h of treatment. This effect is achieved by specifically binding to the N-terminal region of α-syn, destabilizing its β-sheet structure, and inhibiting its pathological aggregation (Minyan, 2016).

**FIGURE 2:**
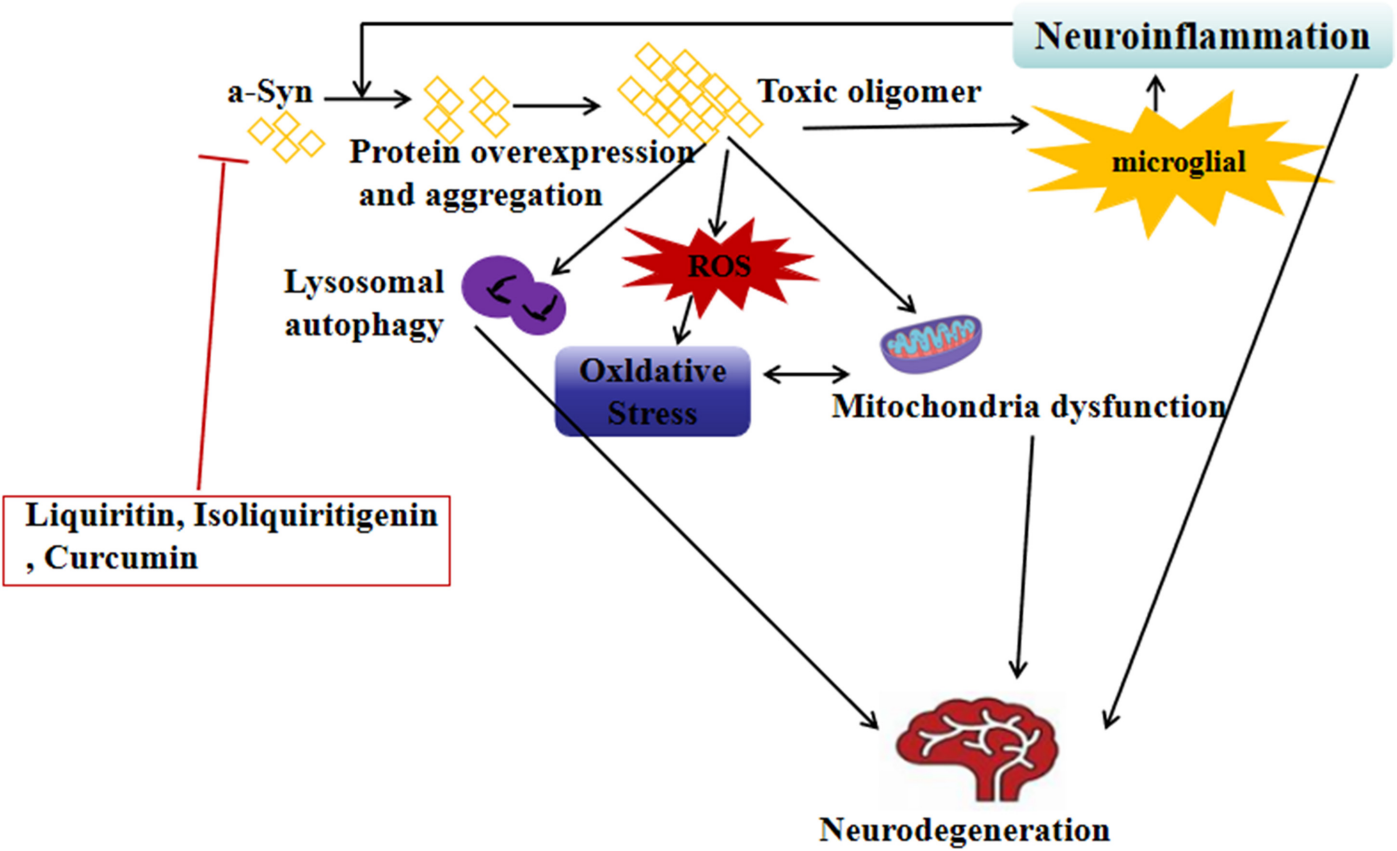
Neuroprotective plant extracts inhibit excessive aggregation of α-synuclein. ⊥, inhibit; ↓, reducing; α-syn, Anti-α-synuclein; ROS, reactive oxygen species.

Curcumin, the primary active component of *Curcuma longa* L., exhibits multi-target neuroprotective effects in MPTP-induced PD rat models. Studies show that curcumin intervention (30 mg/kg/day for 28 days) significantly alleviates motor dysfunction in PD model rats, prolonging the latency period in the rotarod test. Regarding neuroprotective mechanisms, curcumin reduces dopaminergic neuronal toxicity and increases the number of TH-positive neurons in the substantia nigra pars compacta. It also enhances autophagy by upregulating the LC3-II/LC3-I ratio and Beclin-1 protein expression, while promoting α-syn clearance and reducing striatal α-syn aggregates. Molecular studies suggest that curcumin regulates autophagy by activating the AMPK/mTOR signaling pathway (increased p-AMPK expression) and suppresses neuroinflammation by inhibiting the NF-κB pathway (decreased p-p65 expression) (Zhijin and Jianqin, 2024). From a structure-activity relationship perspective, the phenolic hydroxyl group and its adjacent methoxy group in curcumin provide the structural basis for its antioxidant capacity and are critical for inhibiting the excessive aggregation of α-syn (Figure 2).

Extensive research suggests that neuroprotective plant extracts exert their effects in PD animal models through multi-target mechanisms, including the inhibition of abnormal α-syn aggregation, promotion of its clearance, and alleviation of neuroinflammation and oxidative stress. Despite showing potential in preclinical studies, further validation is needed regarding the specific molecular targets, bioavailability, and long-term safety of these plant extracts.

### 3.3 Resist mitochondrial dysfunction and inhibit dopaminergic neuron apoptosis

Mitochondrial dysfunction plays a pivotal role in the pathogenesis of PD. The degeneration of dopaminergic neurons in the striatum serves as a primary pathological hallmark of the disease. These neurons, which possess highly specialized morphological features and high bioenergetic demands, are particularly susceptible to mitochondrial dysfunction. On one hand, impaired mitochondrial function results in an inadequate energy supply to dopaminergic neurons, leading to neuronal dysfunction (Pant et al., 2024). On the other hand, mitochondrial dysfunction causes excessive ROS release, damaging mitochondrial membranes, activating apoptotic signaling pathways, and reducing dopamine neurons, thus promoting the onset and progression of PD (Shu-Min et al., 2018).

A study demonstrated that *Eleutherococcus senticosus* extract (EAS) exhibited significant neuroprotective effects in an MPTP-induced PD mouse model. Behavioral assessments showed that mice in the EAS treatment group (45.5 mg/kg/day, administered for 20 days) exhibited significant improvements in motor function, including prolonged latency in the rotarod test and enhanced scores in the suspension test. Transmission electron microscopy confirmed that EAS effectively protected midbrain mitochondrial ultrastructure, increasing mitochondrial cristae density and enhancing ATP production. Molecular mechanism studies revealed that EAS upregulates PGC-1α expression, promotes mitochondrial biogenesis, and reduces ROS generation. These findings elucidate how *Eleutherococcus* extract improves PD symptoms by protecting mitochondrial structure and function (Shu-Min et al., 2018).

Ginsenoside Rc demonstrates significant neuroprotective effects in MPTP-induced PD mouse models. Behavioral analysis revealed that mice in the ginsenoside Rc treatment group (20 mg/kg/day for 14 days) exhibited marked improvements in motor function, including prolonged latency in the rotarod test and increased spontaneous motor activity. At the molecular level, Western blot analysis showed that ginsenoside Rc effectively modulated the expression of apoptosis-related proteins. It significantly downregulated pro-apoptotic proteins Bax and caspase-3, while upregulating the anti-apoptotic protein Bcl-2, thereby reducing mitochondrial Cyt C release. Transmission electron microscopy further confirmed that ginsenoside Rc preserved mitochondrial structural integrity and significantly improved mitochondrial membrane potential, as evidenced by an increased JC-1 red-to-green fluorescence ratio. These findings suggest that ginsenoside Rc protects dopaminergic neurons by inhibiting the mitochondrial-mediated apoptosis pathway (Zhao et al., 2024). From a structure-activity relationship perspective, Rc, a protopanaxatriol-type ginsenoside, may regulate the mitochondrial apoptosis pathway through its aglycone structure.

**FIGURE 3:**
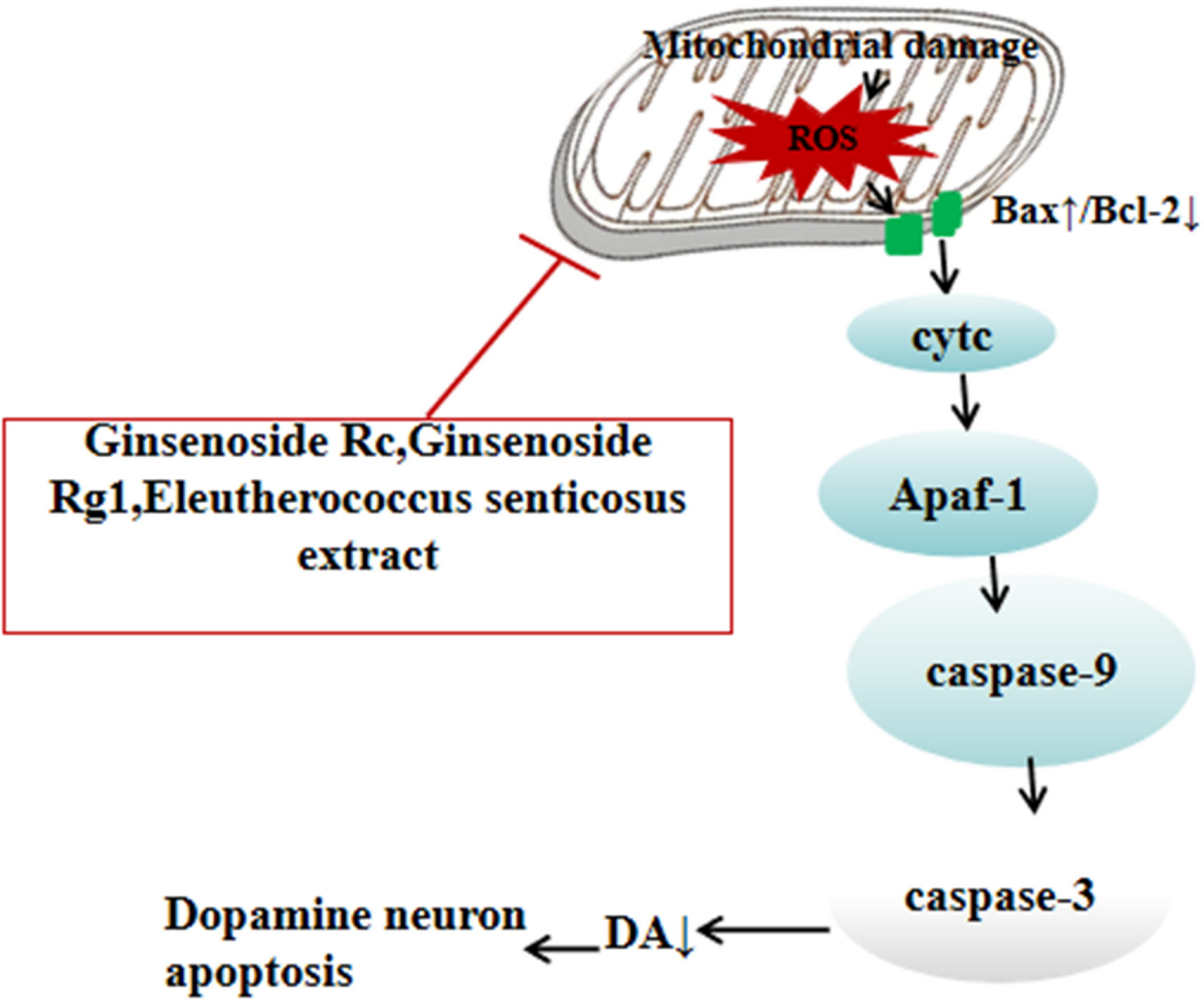
Neuroprotective plant extracts against mitochondrial dysfunction. ⊥, inhibit; ↓, reducing; ROS, reactive oxygen species; Bcl-2, B-cell lymphoma 2; Bax, BCL2-associated X protein; cytc, cytochrome c; Apaf-1, apoptotic protease activating factor-1; caspase-3, cysteine-aspartic acid protease 3; caspase-9, cysteine-aspartic acid protease 9; DA, dopamine.

Apoptosis of dopaminergic neurons in the substantia nigra is a key factor influencing the progression of PD. The B-cell lymphoma 2 (Bcl-2) gene, an effector molecule downstream of the phosphoinositide 3-kinase (PI3K)/protein kinase B (Akt) signaling pathway, exerts potent anti-apoptotic effects by regulating mitochondrial membrane permeability. Studies show that in MPTP-induced PD mouse models, treatment with ginsenoside Rg1 (10 mg/kg/day for 3 days) significantly improves motor dysfunction, with prolonged latency in the rotarod test and enhanced suspension scores. Mechanistic studies suggest that Rg1 reduces caspase cascade activation by modulating apoptosis-related proteins (e.g., upregulating Bcl-2 and suppressing Bax and p-c-Jun expression). Additionally, Rg1 indirectly inhibits apoptosis by enhancing antioxidant defenses (e.g., elevating GSH levels in the substantia nigra) and suppressing neuroinflammation (e.g., inhibiting NF-κB pathway activation and downregulating inflammatory factors like TNF-α and IL-6). These combined effects result in an increase in the number of TH^+^ neurons in the substantia nigra (Shuxiu et al., 2015; Xianchang, 2016; Figure 3).

### 3.4 Anti-inflammatory effects

The pathogenesis of PD involves immune and neuroinflammatory mechanisms. While immune and neuroinflammatory responses are protective against infection and injury, excessive activity in these pathways can damage normal tissues, leading to inflammatory injury of dopaminergic neurons and driving PD progression (Mesarosova et al., 2024). Microglia-mediated neuroinflammation plays a critical role in PD pathogenesis. Abnormal microglial activation promotes the release of pro-inflammatory cytokines, exacerbating dopaminergic neuron damage. Additionally, astrocytes contribute significantly to PD progression, and their dysfunction may reduce neuroprotective capacity, further intensifying neuroinflammatory responses (Sadegh et al., 2023). Extensive research has identified NLRP3 inflammasome activation as a key link between neuroinflammation and dopaminergic neuronal death (Jia et al., 2024). NLRP3 inflammasome activation has been observed in numerous patients with PD, and its activation level correlates with the severity of clinical manifestations (Al-Abbasi, 2022). Suppressing NLRP3 expression improves motor dysfunction, reduces dopaminergic neuron damage, and alleviates microglial activation.

In MPTP-induced PD mouse models, astragaloside IV (AS-IV), the primary active component of *Astragalus membranaceus* (Fisch.), administered at doses of 1.5, 3, and 6 mg/kg for 10 days, demonstrated dose-dependent improvements in motor dysfunction. *In vitro* studies showed that AS-IV (10, 20, 40 μM, pretreated for 2 h) significantly protected against MPP^+^-induced astrocyte apoptosis without significant cytotoxicity. Mechanistic studies revealed that AS-IV inhibits JNK signaling pathway activation by downregulating phosphorylated JNK (p-JNK) levels, thereby modulating downstream apoptosis-related molecules, such as reducing the Bax/Bcl-2 ratio and suppressing caspase-3 activation, ultimately blocking the mitochondrial apoptosis pathway. In a model of MPP^+^-stimulated BV2 microglial cell injury, AS-IV suppressed NLRP3 inflammasome activation and caspase-1 activation. Reversal experiments confirmed that suppression of NLRP3 expression significantly attenuated the anti-inflammatory effects of AS-IV, indicating that its effects are dependent on the regulation of the NLRP3 pathway. These findings suggest that AS-IV exerts synergistic neuroprotective effects at the multicellular level by simultaneously targeting the JNK apoptotic pathway and the NLRP3 inflammatory pathway (Lei et al., 2017).

Another study demonstrated that atractylenolide-I (ATR-I), the primary active component of *Atractylodes macrocephala* Koidz., exhibits significant neuroprotective effects in PD models. In MPTP-induced PD mouse models, ATR-I (30 mg/kg, administered for 7 days) significantly improved motor dysfunction and increased the number of TH-positive neurons. Mechanistic studies indicate that ATR-I exerts anti-inflammatory effects by regulating NLRP3 inflammasome-associated signaling pathways: it significantly reduces levels of key pro-inflammatory factors NO and TNF-α, while suppressing expression of inflammatory mediators such as MCP-1 and MMP-9 in microglia, thereby exerting its anti-inflammatory neuroprotective effects (More and Choi, 2017).

**FIGURE 4:**
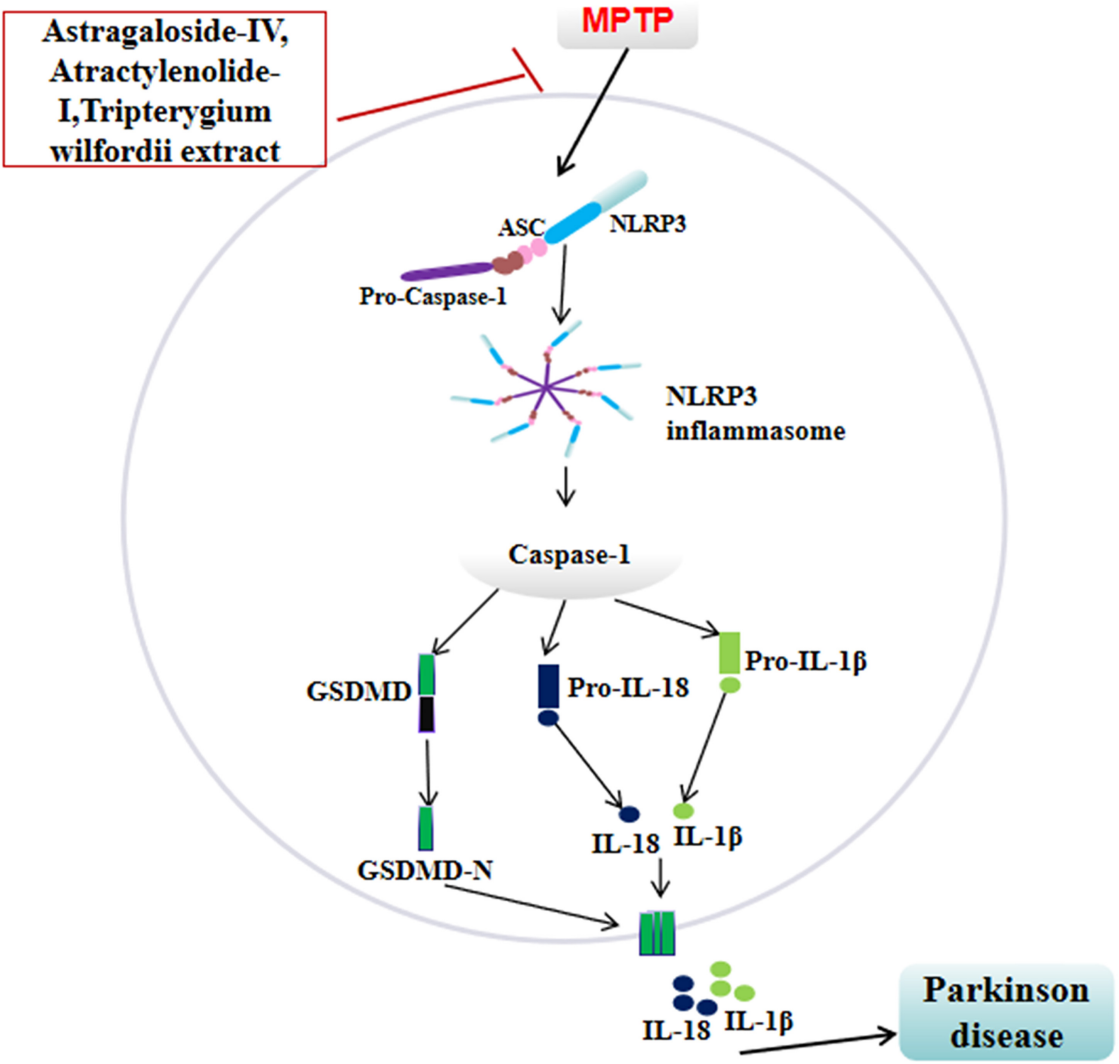
Neuroprotective plant extracts with anti-inflammatory effects. ⊥, inhibit; ↓, reducing; MPTP, mitochondrial permeability transition pore; ASC, apoptosis-associated speck-like protein containing a CARD; NLRP3, NOD-like receptor family pyrin domain containing 3; Caspase-1, cysteine-aspartic acid protease 1; GSDMD, gasdermin D; IL-18, interleukin-18; IL-1β, interleukin-1β.

*Tripterygium wilfordii* extract (10 μg/kg, administered for 7 days) effectively prevents the loss of dopaminergic neurons in the substantia nigra and maintains the function of the nigrostriatal pathway, while significantly improving motor deficits in MPTP-induced PD mouse models. Its mechanism of action follows a clear pathway hierarchy and gene dependency: On one hand, *Tripterygium wilfordii* extract enhances cellular antioxidant defenses by activating the transcription factor Nrf2; on the other hand, it significantly suppresses NLRP3 inflammasome activation and downstream caspase-1 expression, thereby mitigating neuroinflammation. Genetic knockout models provided robust reverse validation: In Nrf2-KO mice, the neuroprotective effects of *Tripterygium wilfordii* extract nearly disappeared, indicating a strict dependence on Nrf2 activation. Furthermore, in NLRP3-KO and caspase-1-KO mice, MPTP-induced pathological phenotypes were significantly attenuated, and the extract failed to provide additional protection. This not only confirms the central role of the NLRP3-caspase-1 pathway in PD pathogenesis but also indicates that *Tripterygium wilfordii* extract exerts its anti-inflammatory effects by inhibiting this pathway. Further investigation revealed minimal impact of *Tripterygium wilfordii* on NLRP3/caspase-1 levels in MPTP-treated Nrf2-KO mice, suggesting that the extract may exert its anti-PD effects by activating Nrf2 to subsequently inhibit the NLRP3 inflammasome (Chenyu et al., 2021; Figure 4).

In summary, the efficacy of neuroprotective plant extracts in preclinical anti-PD studies stems from their multidimensional, systemic intervention across the disease’s complex network. Oxidative stress and mitochondrial damage are causally intertwined; oxidative stress and inflammation mutually amplify each other; and α-synuclein aggregation directly overlaps with organelle dysfunction and neuroinflammation. The diverse bioactive compounds in plant extracts simultaneously target multiple nodes within these pathological networks-activating the Nrf2 pathway for antioxidant defense, suppressing the NF-κB/NLRP3 pathway for anti-inflammation, and intervening in α-synuclein aggregation or enhancing autophagic clearance. This “one target, multiple effects” or “multiple targets synergistically” characteristic enables them to simultaneously disrupt the mutually reinforcing relationships among oxidative stress, neuroinflammation, and protein toxicity. Compared to single-target drugs, this holistic regulatory approach better aligns with PD’s nature as a complex systemic disease, offering novel strategies for future clinical treatment.

### 3.5 Modulating gut-brain axis

First, an imbalance in the gut microbiota directly triggers an inflammatory response in the gut and impairs the intestinal barrier function (Unger et al., 2016). Second, the inflammation that follows barrier damage can cause the enteric nervous system (ENS) to abnormally produce large amounts of α-syn, which may then be transported retrograde to the brain via the vagus nerve, leading to neurotoxicity (Lubomski et al., 2020). In addition, dysbiosis is associated with a reduction in the abundance of short-chain fatty acid (SCFA)-producing bacteria; decreased SCFA levels are closely linked to intestinal inflammation, impaired intestinal barrier function, and central nervous system inflammation. These intestinal pathological changes, by activating the gut-brain axis, may serve as a key driver of the gastrointestinal dysfunction commonly observed in PD patients (particularly the elderly) and contribute to the systemic pathogenesis of PD (Jianfei et al., 2025).

Studies have shown that curcumin (60 mg/kg, administered continuously for 49 days), the primary active component of turmeric (*Curcuma longa* L.), has a beneficial effect on dopaminergic neuron damage and gastrointestinal dysfunction in an MPTP-induced mouse model of Parkinson’s disease (PD). Behavioral results showed that following curcumin treatment, the total distance traveled and average speed of MPTP-treated mice improved significantly, and the loss of TH-positive neurons in the substantia nigra pars compacta (SNpc) and striatum was significantly reduced. Mechanistic studies indicate that curcumin reduces the levels of pro-inflammatory cytokines (IL-1β, IL-6, IL-18, and TNF-α) in intestinal tissues, thereby alleviating intestinal inflammation. It also upregulates the expression of the gastrointestinal barrier proteins ZO-1 and occludin in MPTP-treated mice, improves intestinal barrier function and gastrointestinal dysfunction, and consequently exerts a neuroprotective effect indirectly via the gut-brain axis (Lifan et al., 2022).

Another study demonstrated that administration of neohesperidin, the primary active component of citrus fruits (25 and 50 mg/kg, administered for 24 days), significantly alleviated MPTP-induced damage to dopaminergic neurons in the substantia nigra-striatal pathway of PD mice, increased levels of striatal dopamine (DA) and its metabolite DOPAC, and improved motor dysfunction. Mechanistic studies indicate that, in terms of gut regulation, neohesperidin can modulate MPTP-induced gut microbiota dysbiosis, manifested by increased abundance of beneficial bacteria (Bacteroidetes, Lactobacillaceae) and decreased abundance of harmful bacteria (Actinobacteria, Erysipelotrichaceae, Desulfovibrionaceae, Ruminococcaceae, *Adlercreutzia*, *Allobaculum*, *Oscillospira*, and *Psychrobacter*), reduce serum LPS levels, alleviate colonic tissue damage and the expression of pro-inflammatory factors in the colon, and indirectly suppress neuroinflammation via the gut-brain axis. At the same time, fecal microbiota transplantation (FMT) experiments provided further evidence that neohesperidin combats PD by modulating the gut microbiota; when fecal microbiota from control mice treated with neohesperidin was transplanted into PD mice, it significantly improved motor dysfunction and reduced neuronal damage in the PD mice (Dewei et al., 2024).

### 3.6 Regulate peripheral immunity

There is close interaction between the peripheral immune system and the central nervous system immune system. Inflammatory mediators of peripheral origin (such as cytokines) can enter the central nervous system through permeable regions of the blood-brain barrier or via active transport, where they activate microglia and exacerbate neuroinflammation (Xin et al., 2022).

Studies have shown that resveratrol (RES) (30 mg/kg, once every 3 days for 3 months) improves motor dysfunction and peripheral immune dysregulation in an α-Syn A53T transgenic PD mouse model. Behavioral results showed that after RES treatment, the motor function scores of PD model mice in the pole climbing test, rotarod test, forepaw grip strength test, and four-limb clinging test all improved significantly. Immunological test results indicated that RES significantly increased the proportions of total T lymphocytes and CD4^+^ T cells in the peripheral blood of PD mice, reversing the immune imbalance caused by PD; simultaneously, RES significantly reduced serum levels of the pro-inflammatory cytokines IL-6 and IL-18, while showing a trend toward increased TGF-β levels. Consequently, by inhibiting the cascade amplification of peripheral inflammatory signals to the central nervous system, it indirectly alleviated central nervous system inflammation and exerted a neuroprotective effect (Shenlam et al., 2022).

## 4 Conclusions and outlook

Treatment for PD must address both motor and non-motor symptoms. For a long time, modern medicine has struggled to provide an effective cure for PD, with current treatments only alleviating symptoms, without reversing or halting disease progression. Major neurological disorders like PD arise not from a single large genetic defect but from the interplay of multiple minor genetic factors and environmental influences, collectively forming a complex pathological network (Chenyu et al., 2021). In light of this, neuroprotective plant extracts with multi-target actions present promising strategies for PD intervention. For instance, various active components in ginseng (such as GRe, GRk1, GRc, GRg1, GRg3, and GRb1) simultaneously regulate key pathways, including oxidative stress, mitochondrial dysfunction, neuroinflammation, and apoptosis. Through synergistic mechanisms, these components exert neuroprotective effects, offering greater therapeutic potential intervention than single-target drugs.

This review systematically explores the multi-target mechanisms of neuroprotective plant extracts in PD treatment. These active components help reduce abnormal α-syn aggregation, block its neurotoxic effects, inhibit the apoptosis of dopaminergic neurons in the substantia nigra, regulate microglial activation, reduce the release of pro-inflammatory factors such as TNF-α and IL-1β, enhance antioxidant defense systems (e.g., by upregulating the Nrf2/HO-1 pathway) to mitigate oxidative stress damage, and promote neuroregeneration and synaptic repair. Given these synergistic actions, neuroprotective plant extracts hold significant medicinal potential in PD treatment.

However, the aforementioned conclusions regarding the efficacy of neuroprotective plant extracts are primarily based on data from animal models or cellular experiments. Currently, most existing clinical studies focus on combination regimens of neuroprotective plant extracts and standard anti-Parkinson’s disease medications (such as Madopar and Dopaside tablets); there is still a lack of high-quality, large-scale randomized controlled trials to verify their precise efficacy and underlying mechanisms when used alone. The limitations of the current state of research make discussions regarding the potential toxicity of plant extracts, their long-term safety profiles, and their possible pharmacokinetic/pharmacodynamic interactions with standard medications particularly critical; however, these topics remain a weak point in the field due to insufficient evidence. Given the widespread use of combination therapy in clinical practice, there is an urgent need for well-designed randomized controlled trials to clarify the independent efficacy and safety profiles of herbal extracts, as well as the clinical significance of their interactions with standard treatments. This will provide a reliable basis for transitioning from their current role as adjunctive or complementary therapies to more precise and safer integrated treatment strategies.

Although neuroprotective plant extracts have demonstrated promising efficacy in preclinical studies of Parkinson’s disease, their translation into clinical practice remains fraught with inherent challenges. First, the inherent complexity of the extracts and the lack of clarity regarding their active compounds make it difficult to establish quality control standards. Second, pharmacokinetic limitations-such as low solubility and metabolic instability-are common among the active compounds, and issues with batch-to-batch consistency due to variations in raw material sources represent key bottlenecks hindering their clinical advancement.

At present, sufficient evidence-based medical data is still required to substantiate the therapeutic effects of active components in neuroprotective drugs targeting multiple pathways in PD. Future research can leverage emerging technologies, such as multi-omics integration and spatial metabolomics, to explore molecular-level interactions between signaling pathways and targets. Simultaneously, more clinically relevant studies will offer deeper insights into the targets, pathways, and therapeutic effects of traditional Chinese medicines or compound formulas in PD treatment, providing robust scientific evidence for their application in PD prevention and therapy.
